# Impact of Core-Forming Segment Structure on Drug Loading in Biodegradable Polymeric Micelles Using PEG-*b*-Poly(lactide-*co*-depsipeptide) Block Copolymers

**DOI:** 10.1155/2014/579212

**Published:** 2014-02-20

**Authors:** Akihiro Takahashi, Yuta Ozaki, Akinori Kuzuya, Yuichi Ohya

**Affiliations:** ^1^Organization for Research and Development of Innovative Science and Technology (ORDIST), Kansai University, 3-3-35 Yamate, Suita, Osaka 564-8680, Japan; ^2^Department of Chemistry and Materials Engineering, Faculty of Chemistry, Materials and Bioengineering, Kansai University, 3-3-35 Yamate, Suita, Osaka 564-8680, Japan

## Abstract

We synthesized series of amphiphilic AB-type block copolymers having systematic variation in the core-forming segments using poly(lactide-*co*-depsipeptide)s as a hydrophobic segment and prepared polymeric micelles using the block copolymers, PEG-*b*-poly(lactide-*co*-depsipeptide). We then discussed the relationship between the core-forming segment structure and drug loading efficiency for the polymeric micelles. PEG-*b*-poly(lactide-*co*-depsipeptide)s, PEG-*b*-PLGL containing l-leucine (Leu), and PEG-*b*-PLGF containing l-phenylalanine (Phe), with similar molecular weights and various mole fractions of depsipeptide units, were synthesized. Polymeric micelles entrapping model drug (fluorescein, FL) were prepared using these copolymers. As a result, PEG-*b*-poly(lactide-*co*-depsipeptide) micelles showed higher drug loading compared with PEG-*b*-PLLA and PEG-*b*-PDLLA as controls. The drug loading increased with increase in the mole fraction of depsipeptide unit in the hydrophobic segments. The introduction of aliphatic and aromatic depsipeptide units was effective to achieve higher FL loading into the micelles. PEG-*b*-PLGL micelle showed higher drug loading than PEG-*b*-PLGF micelle when the amount of FL in feed was high. These results obtained in this study should be useful for strategic design of polymeric micelle-type drug delivery carrier with high drug loading efficiency.

## 1. Introduction

**Figure 1:**
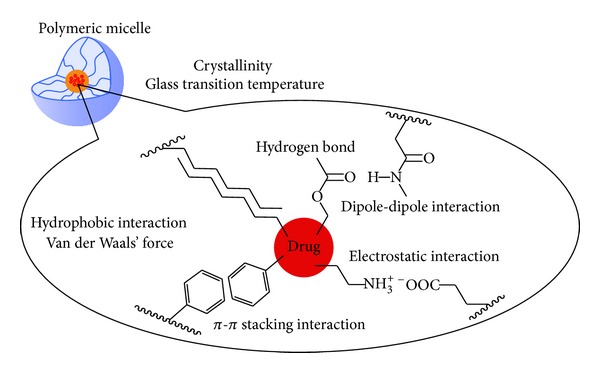
The factors which influence the drug loading efficiency for the polymeric micelle physically entrapping drugs.

Polymeric micelles have attracted much attention in the last two decades as multifunctional nanotechnology-based drug delivery vehicles especially for poorly water-soluble drugs [1–5]. Typically, polymeric micelles are formed by self-aggregation of amphiphilic AB-type diblock copolymers with hydrophobic and hydrophilic segments consisting of inside core and outside shell, respectively. The inner hydrophobic core of a polymeric micelle has a large capacity to accommodate hydrophobic drugs [6], while the hydrophilic shell allows retaining colloidal stability of the polymeric micelle in an aqueous environment [7]. The polymeric micelles are less prone to dissociate even at low concentrations and thus can maintain their micellar structures that facilitate prolonged circulation in the bloodstream by escaping from renal clearance and reticuloendothelial system [8]. The hydrophobic core generally consists of a biodegradable polymer such as poly(*β*-benzyl-l-aspartate) (PBLA) [9], poly(dl-lactide) (PDLLA) [10], and poly(*ε*-caprolactone) (PCL) [11]. A water-soluble polymer may also be used as core-forming segment to render hydrophobicity by the chemical conjugation of a hydrophobic drug [12–14] or polyion complex formation through the association of two oppositely charged polyelectrolytes (polyion complex micelles) [15–17]. Polymeric micelle having hydrophobic core can physically entrap hydrophobic drugs such as doxorubicin (DOX), an anticancer drug, and be used as a carrier to deliver the drugs to a desired site. However, it is not easy to achieve high drug loading and/or entrapment efficiency when the combination of drug and core-forming polymer is not ideal. In fact, some researchers reported the difficulty of physical entrapment of DOX into polymeric micelles [18–22]. To achieve efficient drug delivery to a desired site and to reduce the dose, side effects, and total cost of the formulation, drug loading efficiency is desired to be high enough. The drug loading efficiency strongly depends on solubility of the drug in water (hydrophilicity/hydrophobicity), the preparation method as well as the interaction between the drug and micelle core-forming segment (e.g., hydrogen bonding, hydrophobic interaction, *π*-*π* stacking interaction, dipole-dipole interaction, electrostatic interaction, etc.), and the physical properties of core-forming segment (crystallinity, glass transition temperature, etc.) (Figure 1). However, no systematic study on the relationship between drug loading efficiency and core-forming polymer structure has been carried out.

Polydepsipeptides are copolymers of amino acids and hydroxyl acids and possess degradability of polyesters and functionality of polypeptides. Previously, we reported the synthesis of biodegradable copolymers of lactide and depsipeptide, poly(lactide-*co*-depsipeptide), with reactive side-chain groups such as COOH, NH_2_, OH, and SH by ring-opening copolymerization of lactide with cyclodepsipeptides consisting of the corresponding amino acids and glycolic acid (Glc) [23–26]. Using poly(lactide-*co*-depsipeptide)s we can easily prepare biodegradable copolymers having various properties (hydrophilicity, hydrophobicity, crystallinity, reactivity, electronegativity or -positivity, etc.). So, polydepsipeptide copolymer is one of the most convenient materials for the studies in which systematic variation of the biodegradable polymer properties is needed.

In this study, we synthesized various biodegradable amphiphilic AB-type diblock copolymers of poly(ethylene glycol) (PEG) and poly(lactide-*co*-depsipeptide) containing different amino acids as a hydrophilic segment and a hydrophobic segment, respectively, for preparation of polymeric micelles. We investigated the relationship between the polymer structure and the entrapment behavior of model drugs into the polymeric micelles to optimize the structures of hydrophobic core of the polymeric micelles as drug carriers. We chose l-leucine (Leu) and l-phenylalanine (Phe) as hydrophobic amino acids. PEG-*b*-poly(lactide-*co*-depsipeptide)s were synthesized by ring-opening copolymerization of lactide (LA) with cyclodepsipeptides, cyclo(Glc-Leu), or cyclo(Glc-Phe) using MeO-PEG as a macroinitiator to give PEG-*b*-poly[LA-*co*-(Glc-Leu)] (PEG-*b*-PLGL) and PEG-*b*-poly[LA-*co*-(Glc-Phe)] (PEG-*b*-PLGF). AB-type diblock copolymers of PEG with poly(l-lactide) or poly(dl-lactide), PEG-*b*-PLLA or PEG-*b*-PDLLA, were also synthesized and used as controls. Fluorescein (FL), 4-aminofluorescein (AF), pyrene (PY), and DOX were chosen as model drugs. The structures of these drug model compounds are shown in Supporting Information (see Figure S1 in Supplementary Material available online at http://dx.doi.org/10.1155/2014/579212). Using these copolymers and model drugs, polymeric micelles entrapping the model drugs were prepared by solvent evaporation method. The relationship between the polymer structure, the drug loading, and entrapment efficiency of drugs were then investigated.

## 2. Experimental Section

### 2.1. Materials

Monomethoxy-poly(ethylene glycol) (Mn = 5,000 Da) (MeO-PEG) was purchased from Fluka. Tin 2-ethylhexanoate, PY, and DOX were purchased from Wako Pure Chemical Ind., Ltd. FL and AF were purchased from Tokyo Chemical Industry Co., LTD. Lactides (l- and d,l-isomers) were purchased from Musashino Chemical Laboratory, Ltd. (Tokyo, Japan) and used without further treatment. Organic solvents were purified by usual distillation. Other reagents were of commercial grades and used without further purification.

### 2.2. Measurements

^1^H NMR spectra were recorded on a JNM-GSX-400 (JEOL, 400 MHz) nuclear magnetic resonance instrument using deuterated chloroform (CDCl_3_) as solvent. The chemical shifts were calibrated against TMS and solvent signal of CDCl_3_. The degree of polymerization of lactide and depsipeptide units in hydrophobic segment of the copolymers was calculated based on integral ratios in the ^1^H NMR spectra of PEG-*b*-PLGL and PEG-*b*-PLGF. The number average molecular weight (Mn) and polydispersity index (Mw/Mn) for PEG-*b*-PLGL and PEG-*b*-PLGF were determined by size exclusion chromatography (SEC) (column: TSKgel Multipore HXLM × 2; detector: refractive index) using DMF as an eluent at a flow rate of 1.0 mL/min at 40°C and a series of PEG as standards. Calorimetric analysis was carried out by differential scanning calorimetry (DSC) (Shimadzu DSC-60). The hydrodynamic diameter of micelles was measured by dynamic light scattering (DLS) (Malvern Instruments Ltd. Zetasizer nano Z ZEN 2600). The amounts of encapsulated drug molecules in the micelles were determined by UV-vis absorption spectra in KCl/NaOH solution (for FL and AF) or DMSO (for PY and DOX) using a spectrophotometer (Shimadzu UV-2400PC).

### 2.3. Synthesis of PEG-*b*-Poly(LA-*co*-depsipeptide) Copolymers

**Scheme 1:**
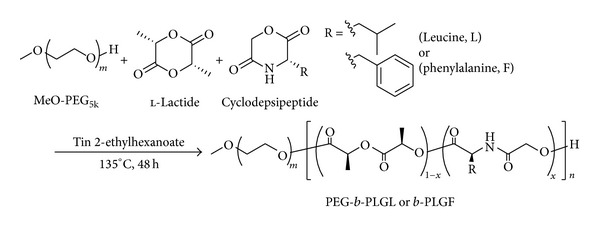
Synthetic route of PEG-*b*-poly(LA-*co*-depsipeptide) copolymers.

A series of PEG-*b*-poly(LA-*co*-depsipeptide) copolymers was synthesized through bulk ring-opening copolymerization of l-LA with cyclo(Glc-Leu) or cyclo(Glc-Phe) using tin 2-ethylhexanoate as a catalyst according to the same method reported previously [23] as shown in Scheme 1. Typical example for PEG-*b*-PLGL is as follows. Under a nitrogen atmosphere, MeO-PEG (400 mg, 80.0 *μ*mol), l-LA (277 mg, 1.90 mmol), and cyclo(Glc-Leu) (329 mg, 1.90 mmol) were placed into a glass tube, followed by the addition of a freshly prepared solution of tin 2-ethylhexanoate (1.60 mg, 3.80 *μ*mol) in anhydrous THF in a glove box. The solvent was removed under vacuum overnight. The tube was then purged with argon and sealed in vacuo. The sealed tube was placed in an oil bath at 160°C for 2 min and then at 135°C for 24 h. The purification of the reaction mixture was performed by the reprecipitation three times using chloroform as a solvent and diethyl ether as a nonsolvent. The obtained precipitate was dried under vacuum overnight to give PEG-*b*-PLGL copolymer with 49% mole fraction of Glc-Leu unit in the PLGL segments (code:*** b*-PLGL**_**49**_) (yield: 86%). ^1^H NMR (400 MHz, CDCl_3_): **δ** = 0.75–1.06 (br, CH_2_CH(CH_3_)_2_), 1.45–1.63 (m, COCH(O)CH_3_), 1.63–1.83 (br, CHCH_2_CH and CH_2_CH(CH_3_)_2_), 3.38 (s, CH_3_O), 3.52–3.80 (m, CH_3_O(CH_2_CH_2_O)_m_CH_2_CH_2_), 4.06–4.91 (br, OCH_2_CH_2_OCO, COCH(CH_2_)NH and NHCOCH_2_O), 5.01–5.26 (m, OCOCH(CH_3_)O).

PEG-*b*-PLGF with 49% mole fraction of Glc-Phe unit in the PLGF segments (code: ***b*-PLGF**_**49**_) was synthesized by the same method described above using cyclo(Glc-Phe) instead of cyclo(Glc-Leu) (yield = 76%). ^1^H NMR (400 MHz, CDCl_3_): **δ** = 1.41–1.66 (m, COCH(O)CH_3_), 3.00–3.35 (m, CHCH_2_C), 3.38 (s, CH_3_O), 3.43–3.75 (m, CH_3_O(CH_2_CH_2_O)_m_CH_2_CH_2_), 4.16–4.97 (br, OCH_2_CH_2_OCO, COCH(CH_2_)NH and NHCOCH_2_O), 4.98–5.28 (m, OCOCH(CH_3_)O), 7.05–7.40 (br, C_6_H_5_).

Other PEG-*b*-PLGL and PEG-*b*-PLGF copolymers (code:*** b*-PLGL**_**x**_ and ***b*-PLGF**_**x**_) with various mole fraction of depsipeptide unit (**x**) were also synthesized by changing feed ratio of cyclodepsipeptide, cyclo(Glc-Leu), or cyclo(Glc-Phe) to l-LA. The feeding amounts and the results of the synthesis were described in supporting information.

### 2.4. Preparation of Drug-Loaded Micelles

Drug-loaded micelles were prepared by a solvent evaporation method. Briefly, a given amount of the copolymer and model drug was dissolved in THF (2 mL) in a glass vial at room temperature. Then, the polymer/drug mixture solution was added dropwise into 10 mL of deionized water under vigorous stirring. After stirring, THF was completely removed under reduced pressure with a rotary evaporator at room temperature to give aqueous micelle solution. To remove insoluble part of drugs, centrifugation (14,000 rpm, 15 min, 2 times) and filtration were carried out. Then, the solution was lyophilized to give powdery drug-loaded micelle.

### 2.5. Characterization of Micelles

Hydrodynamic diameters of micelles were measured by DLS before lyophilization. The amount of drugs was calculated by measurement of the absorbance of drugs using Shimadzu UV-2400PC spectrophotometer. *λ*_max⁡_ for FL, AF, PY, and DOX were 491, 488, 338 and 485 nm, respectively. Polymer recovery (PR), drug loading (DL), entrapment efficiency (EE), and drug recovery (DR) were calculated by the following equations:

(1) PR(%)=(polymer  found)(polymer  in  feed)(w/w)×100,DL(%)=(drug  found)(micelle  found)(w/w)×100,EE(%)=[(drug  found)/(micelle  found)][(drug  in  feed)/(micelle  in  feed)](w/w)(w/w)×100,DR(%)=(drug  found)(drug  in  feed)(w/w)×100.

## 3. Results and Discussion

### 3.1. Synthesis of PEG-*b*-Poly(LA-*co*-depsipeptide) Copolymers

**Figure 2:**
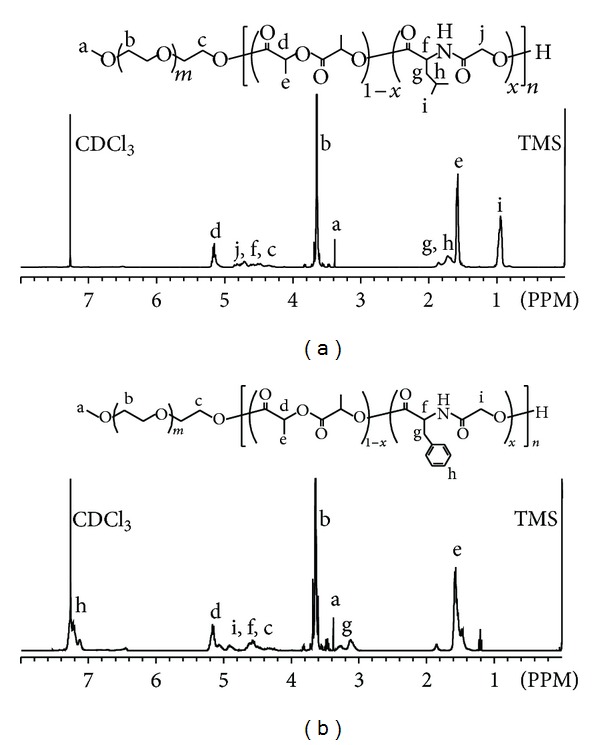
^1^H NMR spectra of (a) PEG-*b*-PLGL (***b*-PLGL**_**49**_) and (b) PEG-*b*-PLGF (***b*-PLGF**_**49**_) in CDCl_3_.

**Figure 3:**
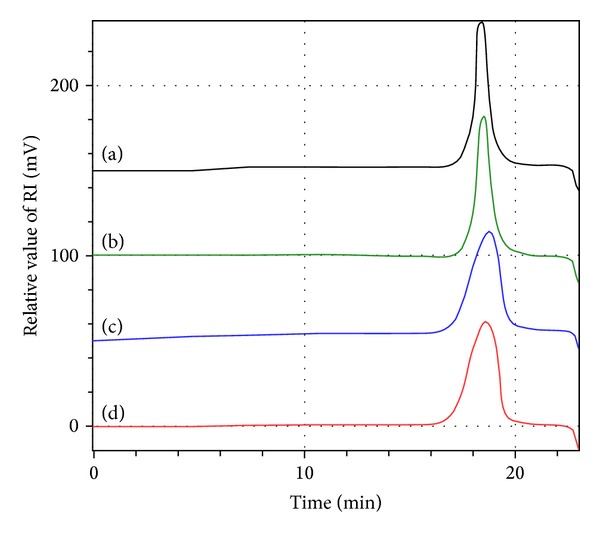
Size exclusion chromatograms of (a) PEG-*b*-PLLA, (b) PEG-*b*-PDLLA, (c) PEG-*b*-PLGL (***b*-PLGL**_**49**_), and (d) PEG-*b*-PLGF_49_ (***b*-PLGF**_**49**_).

PEG-*b*-poly(LA-*co*-depsipeptide) copolymers with various mole fraction of depsipeptide units and similar molecular weights were successfully synthesized. The structures of the copolymers were characterized by ^1^H NMR spectroscopy in CDCl_3_ (Figure 2). The spectrum was given as a simple integration of MeO-PEG and PLGL or PLGF.  Figure 3 shows the results of SEC for PEG-*b*-PLLA, PEG-*b*-PDLLA, PEG-*b*-PLGL, and PEG-*b*-PLGF, and all of the obtained block copolymers showed unimodal molecular weight distribution. The number average molecular weight (Mn) estimated from ^1^H NMR spectra and SEC, degree of polymerization of each hydrophobic segment, mole fraction of depsipeptide unit in each poly (LA-*co*-depsipeptide) segment, and crystallinity estimated by DSC were summarized in Table 1. We use sample codes ***b*-PLLA**, ***b*-PDLLA**, ***b*-PLGL**_**x**_, and ***b*-PLGF**_**x**_ for PEG-*b*-PLLA, PEG-*b*-PDLLA, PEG-*b*-PLGL, and PEG-*b*-PLGF, respectively, and the subscript numbers (**x**) in the codes for ***b*-PLGL**_**x**_ and ***b*-PLGF**_**x**_ mean the mole fraction of depsipeptide units. Mn values for all copolymers estimated from ^1^H NMR spectra were around 1.1 × 10^4^ Da, and these values showed relatively good consistency with the results obtained from SEC measurements. The mole fraction of depsipeptide units in each hydrophobic segment (**x**) was calculated from integration ratio of methine groups of l-LA to methyl groups of Leu or methylene groups of Phe. The crystallinity of hydrophobic segment of copolymers was estimated from a fusion enthalpy measured by DSC. All of PEG-*b*-PLGL and PEG-*b*-PLGF copolymers did not show fusion enthalpy, indicating that the hydrophobic segments in these copolymers were amorphous. On the other hand, PEG-*b*-PLLA showed a fusion enthalpy peak, and the crystallinity of PLLA segment was calculated to be ca. 35%.

### 3.2. Effects of Copolymer Structures on Drug Loading into Micelles

**Figure 4:**
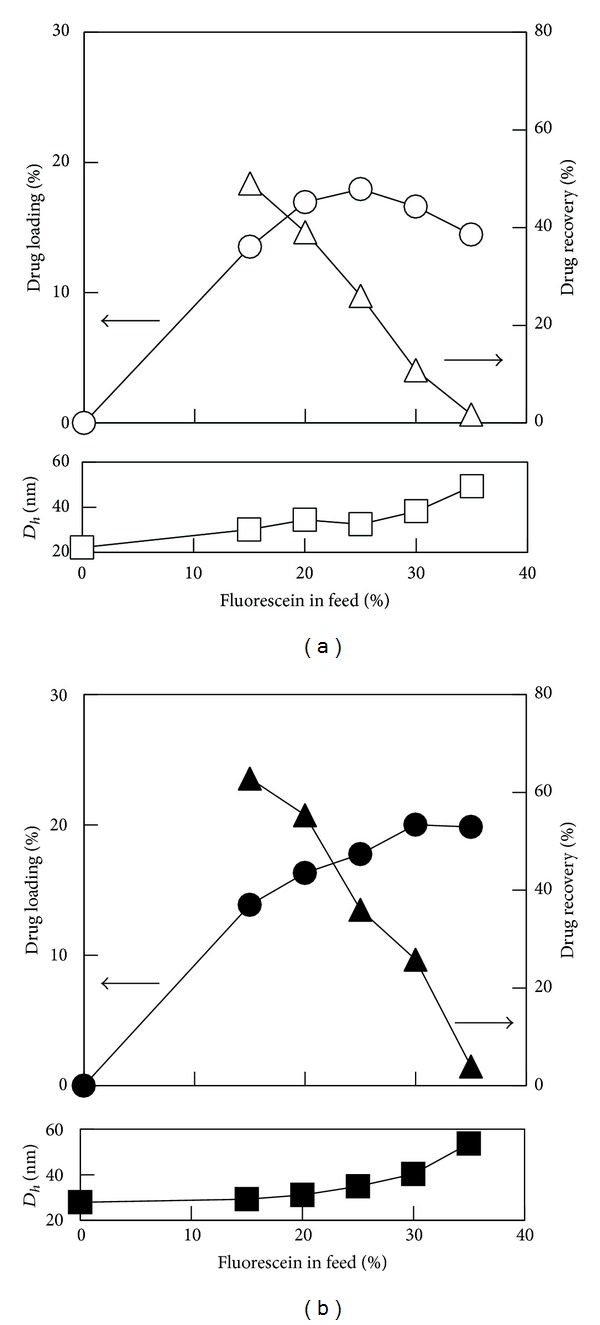
Drug loading (circle), drug recovery (triangle), and hydrodynamic diameter (square) for (a) ***b*-PLLA** and (b) ***b*-PDLLA** micelles versus the amount of fluorescein in feed.

To discuss the relationship between copolymer structures and drug loading behavior, we selected FL, nonionic aromatic fluorophore having hydroxyl groups and cyclic ester group, as a model drug and prepared FL-loaded micelles using various block copolymers with various amount of FL in feed. The results are summarized in Table 2. In Table 2, each polymeric micelle was expressed by code such as ***b*-PLGL**_**x**_ **(Y)**, where **Y** means the amount of model drug in feed (wt% to polymer). As a trend, yields of micelles, drug recovery (DR), and polymer recovery (PR) were decreased with increase in FL in feed. Presumably, much amount of FL inhibits the formation of micelle and the copolymers precipitated together with unloaded FL: coprecipitation of the copolymer and FL occurred. Before discussing the influence of polymer structures on the drug loading behavior, to estimate the effect of crystallinity of hydrophobic segment, the results for semicrystalline ***b*-PLLA** and amorphous ***b*-PDLLA** were discussed.  Figure 4 shows the relationship of DL, DR, and hydrodynamic diameter with FL in feed for ***b*-PLLA** and ***b*-PDLLA** micelles. When the amount of FL in feed was below 25 wt%, DL for ***b*-PLLA** and ***b*-PDLLA** micelles was almost the same and increased with increase in the amounts of FL in feed. However, the DL for ***b*-PLLA** micelle was slightly decreased with increase in the amounts of FL in feed where the amounts of FL in feed were more than 30 wt%. In this condition, ***b*-PDLLA** micelles showed higher DL than ***b*-PLLA** micelle. These results suggest that amorphous state of hydrophobic segments in the core of polymeric micelle is suitable to entrap FL molecules at the high range of feed amount. But the crystallinity does not have significant influence on the drug loading when the feed amount of FL is below 25%. In addition, increase in hydrodynamic diameter of micelles was observed with the increase in feed amount of FL.

**Figure 5:**
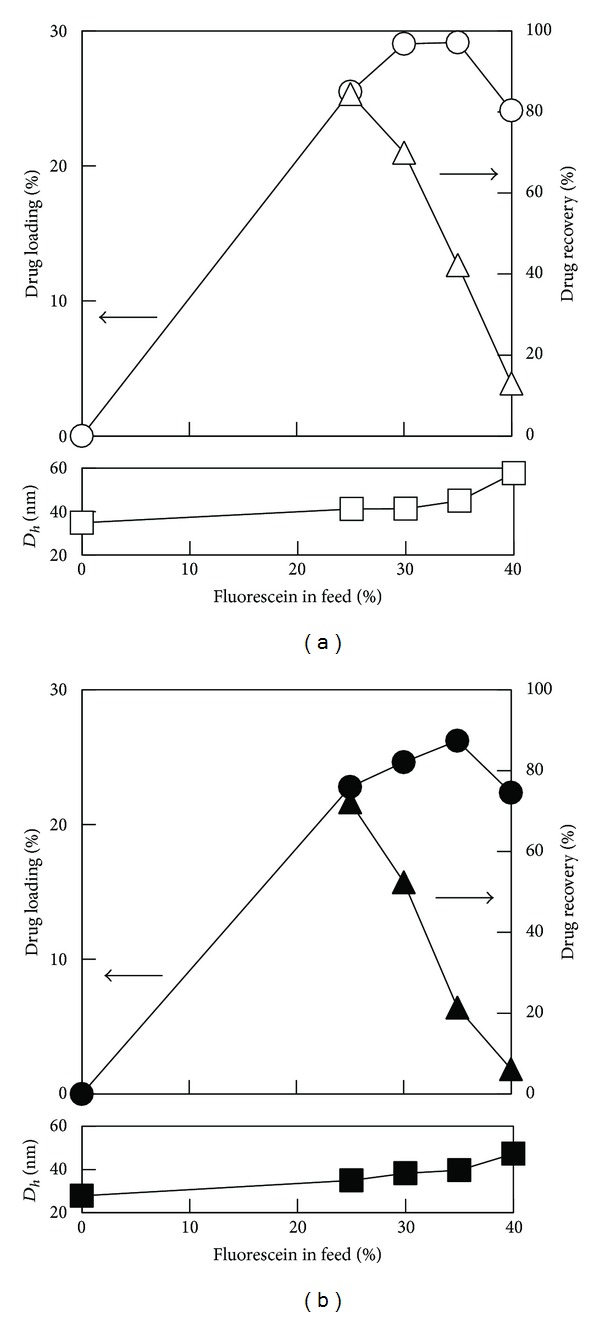
Drug loading (circle), drug recovery (triangle), and hydrodynamic diameter (square) for (a) ***b*-PLGL**_**49**_ and (b) ***b*-PLGF**_**49**_ micelles versus the amount of fluorescein in feed.

The results for ***b*-PLGL** and ***b*-PLGF** micelles were then compared to discuss the effect of side-chain structures of the core-forming segment.  Figure 5 shows the results for ***b*-PLGL**_**49**_ and ***b*-PLGF**_**49**_ micelles as typical examples. Both of ***b*-PLGL** and ***b*-PLGF** micelles showed similar tendency and higher DL values compared with ***b*-PLLA** and ***b*-PDLLA** micelles. DL increased with increase in the amount of FL in feed until the feed amount was 35 wt%, but decreased at the high feed amount of FL (40%). ***b*-PLGL** micelle showed slightly higher DL value compared with ***b*-PLGF** micelle.

**Figure 6:**
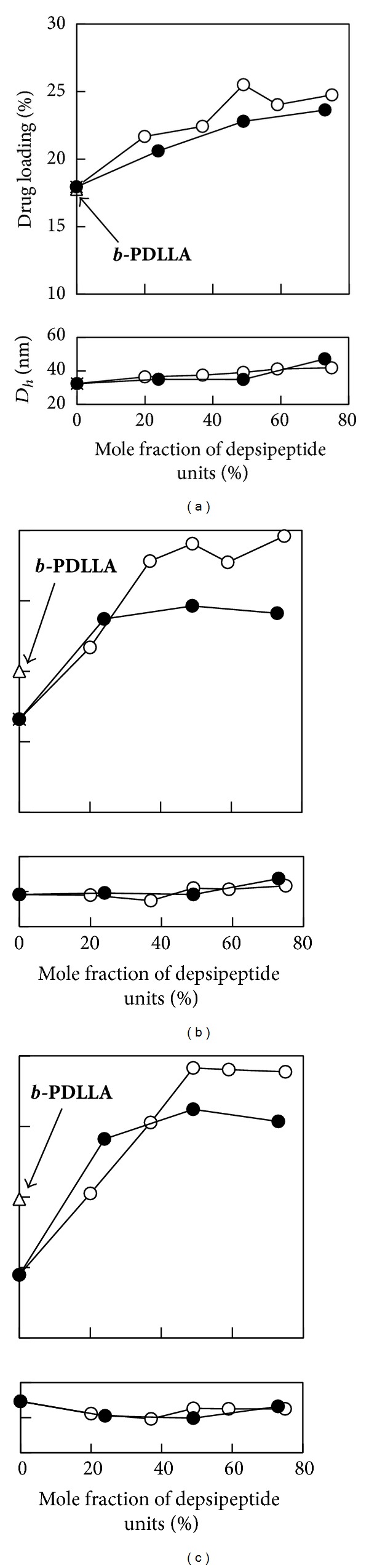
Drug loading and hydrodynamic diameter for ***b*-PLGL** (open circle) and ***b*-PLGF** (closed circle) micelles versus mole fraction of depsipeptide units at various fluorescein in feed (a) 25%, (b) 30%, and (c) 35%. The values for ***b*-PDLLA** micelle were shown as open triangles.

To discuss the effect of side-chain structure of core-forming segment in detail, the dependence of DL and hydrodynamic diameter on the mole fraction of depsipeptide units for ***b*-PLGL** and ***b*-PLGF** micelles was summarized in Figure 6. In Figures 6(a), 6(b), and 6(c), the plots at which mole ratio for depsipeptide units is 0% mean the results of ***b*-PLLA** micelles, and the results of ***b*-PDLLA** micelles were shown as open triangles. ***b*-PLGL** and ***b*-PLGF **micelles showed higher DL compared with ***b*-PLLA** and ***b*-PDLLA** micelles at the ranges of the amount of FL feed (30–40 wt%). In addition to the decrease in crystallinity of the hydrophobic segments, the introductions of aliphatic (Leu) and aromatic (Phe) side chains had positive influence on DL of the micelles. Additionally, the presence of amino bonds might have some positive influence on the drug loading behavior for these micelles. In Figure 6(a) (the amount of FL in feed = 25 wt%), the DL for ***b*-PLGL** micelles increased with increase in the mole fraction of depsipeptide units until they reached 49%, but plateaued where the mole fraction of depsipeptide units was over 50%. In Figures 6(b) and 6(c) (the amounts of FL in feed = 30 and 35 wt%) showed similar trend for DL versus mole fraction of depsipeptide units. ***b*-PLGF** micelles showed a similar tendency in the relationship of DL and mole fraction of depsipeptide units as ***b*-PLGL** micelles showed. Interestingly, the maximum DL values in each figure for ***b*-PLGL** micelles were higher than those for ***b*-PLGF** micelles. Before the experiments, we presumed that ***b*-PLGF** with aromatic side chains would show higher DL value than aliphatic ***b*-PLGL** because of *π*-*π* stacking interaction of benzyl groups of PLGF segment and aromatic FL molecules. But the results were reverse. The reason has not been explained. Based on these results, the miscibility of FL with PLGL segments is higher than PLGF. Flexibility and segmental dynamics of PLGL in the micelle core can presumably be favorable to uptake larger amounts of FL molecules compared with PLGF having aromatic side chains.

Finally, we discussed the combination of core-forming segment and drugs (Table 3). AF was chosen to discuss the effect of polar (amino) group on DL for the micelles. As a result, DL values of AF for all polymeric micelles tested were significantly smaller than those of FL. But the DL values of AF for ***b*-PLGL** and ***b*-PLGF** micelles were larger than those for ***b*-PLLA** and ***b*-PDLLA** micelles, showing similar trends as the cases of FL. These results mean that small difference in drug molecule structure has great influence on the drug loading into the micelles. Probably relatively high water solubility of AF had critical influence on the entrapment behavior of the model drug into the micelles. On the other hand, PY and DOX gave contrastive results. Both of PY and DOX are hydrophobic poorly water-soluble aromatic compounds, but DOX has polar hydroxyl and amino groups. PY consisting only of aromatic ring was hardly loaded into all micelles tested, and the DL values were around 0.6%. However, the PR of the PY-loaded micelles was relatively high (78–87%) indicating similar trends observed for AF. On the other hand, DOX showed high level DL values (around 30%) and high PR (83–89%) for all of the micelles tested. Although DL of FL into ***b*-PLGL** and ***b*-PLGF** micelles were higher than those into ***b*-PLLA** and ***b*-PDLLA** micelles, the DL values of DOX for all of the micelles were not significantly different. These all results mean that the combination of core-forming polymer and drugs has critical influence on the drug loading behavior into polymeric micelles.

## 4. Conclusions

In this study, we discussed the relationship between the structure of core-forming segment and the structure of drugs to optimize the biodegradable AB-type amphiphilic block copolymer for polymeric micelle-type drug delivery carriers. Using PEG-*b*-poly(LA-*co*-depsipeptide), the systematic variation of core-forming (hydrophobic) segments could be provided. PEG-*b*-PLGL and PEG-*b*-PLGF with the similar molecular weights and various mole fractions of depsipeptide units were synthesized and used for the preparation of polymeric micelles entrapping model drug (FL). The yields of micelles and a DR were decreased with the increase in the amounts of FL in feed. From the results for PEG-*b*-PLLA and PEG-*b*-PDLLA, noncrystallinity of the hydrophobic segments was suitable to load the FL where the amount of FL in feed was high. Both of PEG-*b*-PLGL micelle with aliphatic (Leu) side-chain groups and PEG-*b*-PLGF micelle with aromatic (Phe) side-chain groups showed higher DL values compared with PEG-*b*-PDLLA micelles. The introduction of aliphatic or aromatic side-chain groups was effective to increase the drug loading, and DL values for PEG-*b*-PLGL and PEG-*b*-PLGF were increased with increasein the mole fraction of depsipeptide units. PEG-*b*-PLGL micelle showed higher DL than PEG-*b*-PLGF micelle only when the amount of FL in feed was high. The interaction of aromatic side chain of PLGF segment with aromatic model drug compound had no significant impact on the drug loading. Comparing the four model drugs (FL, AF, PY, and DOX) used in this study, it was revealed that the combination of the core-forming segments and drugs had great influences on DL and PR. All information obtained in this study should be useful for rational design of polymeric micelle-type drug delivery carrier with high drug loading efficiency.

## Supplementary Material

Supplementary Material includes the synthetic results of the other block copolymers and the structures of drug model compounds.

## Figures and Tables

**Table 1:** Results of characterization of copolymers.

Code	***w*** ^ a^ [mol%]	Mn^b^(×10^−4^)	Mn^c^ (×10^−4^)	Mw/Mn^c^	DP^b,d^	**x** ^ b,e^ [mol%]	*X*_*c*_^f^ [%]
***b*-PLLA**	—	1.15	0.90	1.27	45	—	35
***b*-PDLLA**	—	1.15	0.81	1.29	45	—	—
***b*-PLGL**_20_	20	1.09	0.92	1.35	40	20	0
***b*-PLGL**_37_	40	1.13	1.03	1.35	40	37	0
***b*-PLGL**_49_	50	1.11	0.90	1.38	39	49	0
***b*-PLGL**_59_	60	1.24	1.11	1.42	46	59	0
***b*-PLGL**_75_	75	1.17	1.02	1.39	41	75	0
***b*-PLGF**_24_	24	1.06	0.91	1.38	34	24	0
***b*-PLGF**_49_	50	1.11	0.91	1.38	35	49	0
***b*-PLGF**_73_	68	1.10	0.89	1.42	32	73	0

^a^Mole fraction of depsipeptide units in feed. ^b^Estimated by ^1^H NMR (solvent: CDCl_3_). ^c^Estimated by SEC (eluent: DMF; standard: PEG). ^d^Degree of polymerization of sum of lactide and depsipeptide units. ^e^ **x**: mole fraction of depsipeptide units in a hydrophobic segment of diblock copolymers. ^f^ *X*_*c*_: crystallinity determined by differential scanning calorimetry (DSC). *X*_*c*_ = (Δ*H*_*m*_ + Δ*H*_*c*_)/Δ*H*_*m*, theory_ (Δ*H*_*m*, theory_ = −93.7 J/g).

**Table 2:** Results of preparation of fluorescein-loaded polymeric micelles.

Code	Fluorescein in feed [wt%]	Yield mg (%)	PR [%]	DL [%]	EE [%]	DR [%]
***b*-PLLA(0)**	0	21.0 (84)	84	0.0	—	—
***b*-PLLA(10)**	10	16.7 (60)	60	10.4	104	63
***b*-PLLA(15)**	15	16.0 (54)	55	13.5	90	49
***b*-PLLA(20)**	20	14.4 (46)	48	16.9	85	39
***b*-PLLA(25)**	25	12.1 (36)	40	17.9	72	26
***b*-PLLA(30)**	30	7.0 (20)	23	16.6	60	11
***b*-PLLA(35)**	35	1.6 (4)	5	14.5	41	1.7

***b*-PDLLA(0)**	0	22.3 (89)	89	0.0	—	—
***b*-PDLLA(10)**	10	22.3 (80)	79	11.6	116	93
***b*-PDLLA(15)**	15	20.0 (68)	69	13.9	92	63
***b*-PDLLA(20)**	20	21.2 (68)	71	16.3	82	55
***b*-PDLLA(25)**	25	16.9 (51)	56	17.8	71	36
***b*-PDLLA(30)**	30	13.8 (39)	44	20.0	67	26
***b*-PDLLA(35)**	35	2.7 (7)	9	19.9	57	4.0

*** b*-PLGL**_**20**_ **(0)**	0	22.0 (89)	89	0.0	—	—
***b*-PLGL**_20_ **(25)**	25	24.9 (75)	78	21.7	87	65
***b*-PLGL**_20_ **(30)**	30	23.1 (65)	72	21.7	72	47
***b*-PLGL**_20_ **(35)**	35	12.9 (34)	41	20.2	58	19
***b*-PLGL**_20_ **(40)**	40	5.2 (12)	16	22.0	55	6.9

***b*-PLGL**_37_ **(0)**	0	20.4 (82)	82	0.0	—	—
***b*-PLGL**_37_ **(25)**	25	26.8 (80)	83	22.4	90	72
***b*-PLGL**_37_ **(30)**	30	29.7 (83)	86	27.8	93	77
***b*-PLGL**_37_ **(35)**	35	18.9 (49)	56	25.3	72	36
***b*-PLGL**_37_ **(40)**	40	6.6 (16)	20	25.6	64	10

***b*-PLGL**_49_ **(0)**	0	20.7 (83)	83	0.0	—	—
***b*-PLGL**_49_ **(25)**	25	27.6 (83)	82	25.5	102	85
***b*-PLGL**_49_ **(30)**	30	25.8 (72)	73	29.0	97	70
***b*-PLGL**_49_ **(35)**	35	19.5 (51)	55	29.2	83	42
***b*-PLGL**_49_ **(40)**	40	9.0 (22)	27	24.1	60	13

***b*-PLGL**_59_ **(0)**	0	22.6 (90)	90	0.0	—	—
***b*-PLGL**_59_ **(25)**	25	26.5 (80)	81	24.0	96	76
***b*-PLGL**_59_ **(30)**	30	28.1 (79)	81	27.7	92	73
***b*-PLGL**_59_ **(35)**	35	19.8 (51)	56	29.0	83	43
***b*-PLGL**_59_ **(40)**	40	7.1 (17)	20	29.6	74	12.6

***b*-PLGL**_75_ **(0)**	0	22.8 (91)	91	0.0	—	—
***b*-PLGL**_75_ **(25)**	25	26.9 (81)	81	24.8	99	80
***b*-PLGL**_75_ **(30)**	30	25.7 (72)	72	29.6	99	71
***b*-PLGL**_75_ **(35)**	35	19.2 (50)	55	28.9	83	41
***b*-PLGL**_75_ **(40)**	40	11.1 (27)	32	28.5	71	19

***b*-PLGF**_24_ **(0)**	0	23.0 (92)	92	0.0	—	—
***b*-PLGF**_24_ **(24)**	25	22.9 (69)	73	20.6	82	57
***b*-PLGF**_24_ **(30)**	30	22.3 (62)	68	23.7	79	49
***b*-PLGF**_24_ **(35)**	35	12.7 (33)	39	24.1	68	23
***b*-PLGF**_24_ **(40)**	40	8.8 (21)	27	22.0	55	12

***b*-PLGF**_49_ **(0)**	0	20.8 (83)	83	0.0	—	—
***b*-PLGF**_49_ **(25)**	25	26.4 (79)	82	22.8	91	72
***b*-PLGF**_49_ **(30)**	30	22.8 (64)	69	24.6	82	52
***b*-PLGF**_49_ **(35)**	35	11.0 (29)	32	26.2	75	21
***b*-PLGF**_49_ **(40)**	40	4.6 (11)	14	22.4	56	6.2

***b*-PLGF**_73_ **(0)**	0	22.3 (89)	89	0.0	—	—
***b*-PLGF**_73_ **(25)**	25	23.4 (70)	71	23.6	95	66
***b*-PLGF**_73_ **(30)**	30	18.6 (52)	56	24.1	80	42
***b*-PLGF**_73_ **(35)**	35	13.4 (35)	40	25.4	72	25
***b*-PLGF**_73_ **(40)**	40	6.4 (15)	18	28.4	71	11

PR (%) = (polymer found)/( polymer in feed) (w/w) × 100.

DL (%) = (drug found)/(micelle found) (w/w) × 100.

EE (%) = [(drug found)/(micelle found)]/[(drug in feed)/(micelle in feed)] (w/w) × 100.

DR (%) = (drug found)/(drug in feed) (w/w) × 100.

**Table 3:** Characterization of micelles versus drugs in 30% feed.

Drug^a^	Drug loading (DL) (wt%)			
	***b*-PLLA**	***b*-PDLLA**	***b*-PLGL**_49_	***b*-PLGF**_49_
Fluorescein	16.6	20.0	29.0	26.2
4-Aminofluorescein	3.0	4.7	7.3	7.2
Pyrene	0.5	0.6	0.6	0.7
Doxorubicin	30.3	28.6	29.7	29.8

DL (%) = (drug found)/(micelle found) (w/w) × 100.

^a^The amount of drug in feed = 30 wt%.
