# Supplementary material for: Impact of Core-Forming Segment Structure on Drug Loading in Biodegradable Polymeric Micelles Using PEG-b-Poly(lactide-co-depsipeptide) Block Copolymers
Source: Biomed Res Int. 2014 Feb 20;2014:579212. doi: 10.1155/2014/579212 (PMC3950643; doi:10.1155/2014/579212)
Supplement: Supplementary file 1 — Supplementary Material includes the synthetic results of the other block copolymers and the structures of drug model compounds. [file 579212.f1.pdf]

Supporting Information

Impact of Core-forming Segment Structure on Drug Loading in Biodegradable Polymeric Micelles Using PEG-*b*-Poly(lactide-*co*-depsipeptide) Block Copolymers

Akihiro Takahashi[1], Yuta Ozaki[2], Akinori Kuzuya[1,2], Yuichi Ohya[1,2]*

Synthesis of PEG-*b*-PLGL$_{20}$ (20% mole fraction of Leu)

PEG (525 mg, 110 μmol), L-LA (484 mg, 3.40 mmol), cyclo(Glc-Leu) (114 mg, 0.800 mmol), tin 2-ethylhexanoate (1.70 mg, 4.20 μmol), Yield = 84%.

^{1}H NMR (400 MHz, $CDCl_3$): $\delta =$ 0.75-1.06 (br, $CH_2CH(CH_3)_2$), 1.45-1.63 (m, $COCH(O)CH_3$), 1.63-1.83 (br, $CHCH_2CH$ and $CH_2CH(CH_3)_2$), 3.38 (s, CH_3O), 3.52-3.80 (m, $CH_3O(CH_2CH_2O)_mCH_2CH_2$), 4.06-4.91 (br, OCH_2CH_2OCO, $COCH(CH_2)NH$ and $NHCOCH_2O$), 5.01-5.26 (m, $OCOCH(CH_3)O$)

Synthesis of PEG-*b*-PLGL$_{37}$ (37% mole fraction of Leu)

PEG (420 mg, 84.0 μmol), L-LA (363 mg, 2.50 mmol), cyclo(Glc-Leu) (284 mg, 1.70 mmol), tin 2-ethylhexanoate (1.70 mg, 4.20 μmol), Yield = 74%.

^{1}H NMR (400 MHz, $CDCl_3$): $\delta =$ 0.75-1.06 (br, $CH_2CH(CH_3)_2$), 1.45-1.63 (m, $COCH(O)CH_3$), 1.63-1.83 (br, $CHCH_2CH$ and $CH_2CH(CH_3)_2$), 3.38 (s, CH_3O), 3.52-3.80 (m, $CH_3O(CH_2CH_2O)_mCH_2CH_2$), 4.06-4.91 (br, OCH_2CH_2OCO, $COCH(CH_2)NH$ and $NHCOCH_2O$), 5.01-5.26 (m, $OCOCH(CH_3)O$)

Synthesis of PEG-*b*-PLGL$_{59}$ (59% mole fraction of Leu)

PEG (350 mg, 70.0 μmol), L-LA (242 mg, 1.70 mmol), cyclo(Glc-Leu) (431 mg, 2.50 mmol), tin 2-ethylhexanoate (1.70 mg, 4.20 μmol), Yield = 72%.

^{1}H NMR (400 MHz, $CDCl_3$): δ = 0.75-1.06 (br, $CH_2CH(CH_3)_2$), 1.45-1.63 (m, $COCH(O)CH_3$), 1.63-1.83 (br, $CHCH_2CH$ and $CH_2CH(CH_3)_2$), 3.38 (s, CH_3O), 3.52-3.80 (m, $CH_3O(CH_2CH_2O)_mCH_2CH_2$), 4.06-4.91 (br, OCH_2CH_2OCO, $COCH(CH_2)NH$ and $NHCOCH_2O$), 5.01-5.26 (m, $OCOCH(CH_3)O$)

Synthesis of PEG-*b*-PLGL$_{75}$ (75% mole fraction of Leu)

PEG (150 mg, 30.0 μmol), L-LA (61.0 mg, 0.42 mmol), cyclo(Glc-Leu) (216 mg, 1.30 mmol), tin 2-ethylhexanoate (0.68 mg, 1.70 μmol), Yield = 76%.

^{1}H NMR (400 MHz, $CDCl_3$): δ = 0.75-1.06 (br, $CH_2CH(CH_3)_2$), 1.45-1.63 (m, $COCH(O)CH_3$), 1.63-1.83 (br, $CHCH_2CH$ and $CH_2CH(CH_3)_2$), 3.38 (s, CH_3O), 3.52-3.80 (m, $CH_3O(CH_2CH_2O)_mCH_2CH_2$), 4.06-4.91 (br, OCH_2CH_2OCO, $COCH(CH_2)NH$ and $NHCOCH_2O$), 5.01-5.26 (m, $OCOCH(CH_3)O$)

Synthesis of PEG-*b*-PLGF$_{24}$ (24% mole fraction of Phe)

PEG (600 mg, 120 μmol), L-LA (552 mg, 3.80 mmol), cyclo(Glc-Phe) (248 mg, 1.20 mmol), tin 2-ethylhexanoate (2.04 mg, 5.00 μmol), Yield = 80%.

^{1}H NMR (400 MHz, $CDCl_3$): δ = 1.41-1.66 (m, $COCH(O)CH_3$), 3.00-3.35 (m, $CHCH_2C$), 3.38 (s, CH_3O), 3.43-3.75 (m, $CH_3O(CH_2CH_2O)_mCH_2CH_2$), 4.16-4.97 (br, OCH_2CH_2OCO, $COCH(CH_2)NH$ and $NHCOCH_2O$), 4.98-5.28 (m, $OCOCH(CH_3)O$), 7. 05-7.40 (br, C_6H_5).

Synthesis of PEG-*b*-PLGF$_{73}$ (73% mole fraction of Phe)

PEG (200 mg, 40.0 μmol), L-LA (103 mg, 0.72 mmol), cyclo(Glc-Phe) (312 mg, 1.50 mmol), tin 2-ethylhexanoate (0.91 mg, 2.20 μmol), Yield = 50%.

^{1}H NMR (400 MHz, $CDCl_3$): δ = 1.41-1.66 (m, $COCH(O)CH_3$), 3.00-3.35 (m, $CHCH_2C$), 3.38 (s, CH_3O), 3.43-3.75 (m, $CH_3O(CH_2CH_2O)_mCH_2CH_2$), 4.16-4.97 (br, OCH_2CH_2OCO, $COCH(CH_2)NH$ and $NHCOCH_2O$), 4.98-5.28 (m, $OCOCH(CH_3)O$), 7. 05-7.40 (br, C_6H_5).

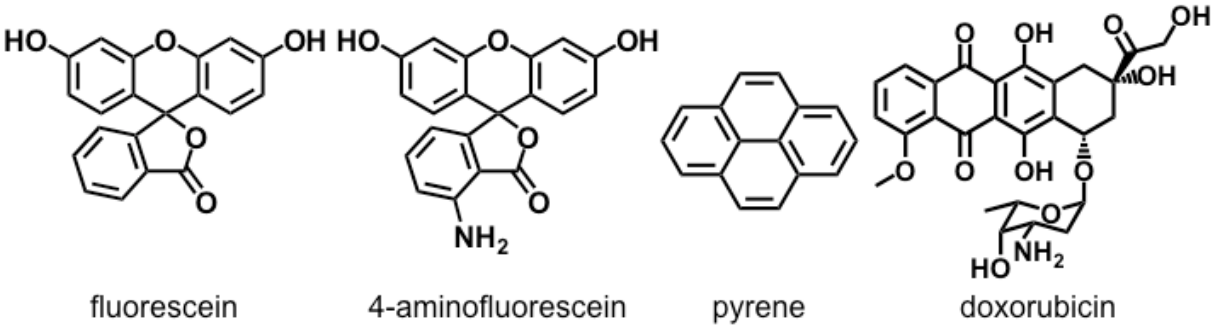

Figure S1. Structure of model drugs used in this study.
